# Lipopolysaccharide potentiates platelet responses via toll-like receptor 4-stimulated Akt-Erk-PLA_2_ signalling

**DOI:** 10.1371/journal.pone.0186981

**Published:** 2017-11-14

**Authors:** Maria E. Lopes Pires, Simon R. Clarke, Sisi Marcondes, Jonathan M. Gibbins

**Affiliations:** 1 School of Biological Science, Institute for Cardiovascular and Metabolic Research, University of Reading, Reading, Berkshire, United Kingdom; 2 Department of Pharmacology, Faculty of Medical Sciences, University of Campinas (UNICAMP), Campinas, SP, Brazil; Freiburg University, GERMANY

## Abstract

Lipopolysaccharide (LPS) from the cell envelope of Gram-negative bacteria is a principal cause of the symptoms of sepsis. LPS has been reported to modulate the function of platelets although the underlying mechanisms of LPS action in these cells remain unclear. Platelets express the Toll-like receptor 4 (TLR4) which serves as a receptor for LPS, although the potential role of TLR4 and associated cell signalling in controlling platelet responses to LPS has not been extensively explored. In this study, we therefore investigated the actions of LPS prepared from different strains of *Escherichia coli* on platelet function, the underlying signalling mechanisms, and the potential role of TLR4 in orchestrating these. We report that LPS increased the aggregation of washed platelets stimulated by thromboxane (U46619) or GPVI collagen receptor agonists, effects that were prevented by a TLR4 antagonist. Associated with this, LPS enhanced fibrinogen binding, P-selectin exposure and reactive oxygen species (ROS) release. Increase of ROS was found to be important for the actions of LPS on platelets, since these were inhibited in the presence of superoxide dismutase or catalase. The effects of LPS were associated with phosphorylation of Akt, ERK1/2 and PLA_2_ in stimulated platelets, and inhibitors of PI3-kinase, Akt and ERK1/2 reduced significantly LPS enhanced platelet function and associated ROS production. Furthermore, inhibition of platelet cyclooxygenase or the thromboxane receptor, revealed an important role for thromboxane A2. We therefore conclude that LPS increases human platelet activation through a TLR4-PI3K-Akt-ERK1/2-PLA_2_ -dependent pathway that is dependent on ROS and TXA_2_ formation.

## Introduction

Platelets are required for haemostasis and respond to tissue injury to form an aggregate or platelet plug that limits the loss of blood. Inappropriate activation of platelets in diseased blood vessels is, however, a major trigger for thrombosis and platelets are increasingly implicated in inflammation and the symptoms of sepsis [1]. Lipopolysaccharide (LPS) from the cell envelope of Gram-negative bacteria is a principal cause of the symptoms of sepsis and this can result in platelet sequestration in the lungs and liver, thrombocytopenia and disseminated intravascular coagulation (DIC) [2, 3]. LPS has been reported to modulate the function of platelets [4, 5, 6], although the underlying mechanisms of LPS action remain unclear. Indeed, some studies have indicated that exposure of platelets to LPS *in vitro* potentiates platelet function [7, 8], while others have indicated that it supresses their activity [9]. Furthermore, LPS from different sources and at distinct concentrations are able to promote variable effects in a range of cells types [10,11].

LPS has been demonstrated to induce excessive secretion of pro-inflammatory mediators such as cytokines, tumor necrosis factor alpha (TNFα) and reactive oxygen species (ROS) in different cells [5, 12], and overproduction of ROS has been associated with damage to vascular endothelium and multiple organ dysfunction [13]. Previous studies have suggested that stimulated and unstimulated platelets are capable of releasing ROS that participate in the control of platelet function [14, 15]. The mechanisms that lead the release of ROS by platelets are, however, incompletely understood.

Platelets, express the pathogen recognition receptor, Toll-like receptor 4 (TLR4) that serves as a receptor for LPS on a range of cells types [3, 16, 17]. The role in platelets that this receptor may play in stimulating the signalling that controls platelet responses to LPS has yet to be explored. Therefore, in the present study we sought to explore the signalling pathway(s) through which LPS modulates platelets regulation.

Ligation of TLR4 induces recruitment of multiple adaptor proteins such as TRIF, MyD88, TIRAP, TRAM and SARM through interactions with Toll-interleukin-1 receptor domains [18]. Previous studies have identified a number of proteins implicated in TRIF dependent signalling (eg. TBK-1, IRAK-1, JNKs, MAPK, TRAF3, TRAF6, IRF-3, Ikk-I, IκB-α, NK-κB) that are present in platelets [7, 19, 20]. In vascular smooth muscle, LPS- mediated signalling promotes activation of the mitogen-activated kinases (MAPKs) p38, ERK1/2 and JNK1/2 and also the phosphoinositide- 3 kinase (PI3-kinase) dependent signalling [21]. Furthermore, Brown and McIntyre showed that in platelets, MyD88, TRAF6, JNK and AKT are required in IL-1β production stimulated by LPS [22].

Platelet activation by agonists such as collagen or thromboxane A_2_ is associated with the activation of a complex network of cell signalling [23, 24] in which several molecules implicated in TLR4 signalling are critically involved, including PI3-kinase (PI3K), Akt, and ERK1/2 [25, 26, 27]. While these signalling proteins have been shown to mediate some actions of LPS on different cells [25, 26], their potential involvement in TLR4 signalling in platelets has not been established.

The aim of this study was to elucidate whether components of the TLR4 signalling pathway are implicated in the functional responses of platelets to LPS and the involvement of ROS production in these responses. Here, we found that LPS increases human platelet activation through a TLR4-PI3K-Akt-ERK1/2-PLA_2_-dependent pathway that is dependent on ROS and TXA_2_ formation.

## Materials and methods

### Isolation and purification of Lipopolysaccharide from Gram-negative bacteria

LPS from *E*. *coli* K12 was isolated and purified using methodology described by Davis and Goldberg, 2012 [28]. The purity LPS samples prepared were visualized by direct staining following separation on 12% SDS-polyacrilamide gel using Pro-Q Emerald 300 Lipopolysaccharide Gel Stain Kit (Sigma Aldrich, UK) (S1 Fig). LPS purified from *E*. *coli* O111:B4 was purchased from Sigma Aldrich.

### Platelet preparation

Blood was drawn from healthy donors that had given informed consent. The study and procedures for obtaining informed consent were approved by the University of Reading Research Ethics Committee. Blood was collected into 50mL syringes containing 4% (w/v) sodium citrate and acid citrate dextrose (ACD, 2.5% sodium citrate, 2% D-glucose and 1.5% citric acid). Platelet preparation was according to the methodology developed previously to limit the levels of leukocyte or erythrocyte contamination to no more than one contamination cell per 6500 platelets [29]. The blood was centrifuged for 20 min at 102× g at room temperature to obtain platelet-rich plasma (PRP). Further centrifugation of PRP [with 125 ng·mL^–1^ prostaglandin I_2_ (PGI2)] at 1413× g for 10 min resulted in a platelet pellet that was re-suspended in modified Tyrodes-HEPES buffer (134 mM NaCl, 2.9 mM KCl, 0.34 mM Na2HPO4·12H_2_O, 12 mM NaHCO3, 20 mM HEPES and 1 mM MgCl_2_, pH 7.3) and washed by centrifuging in the presence of PGI2 again at the same speed for 10 min. The resulting platelet pellet was re-suspended in modified Tyrodes-HEPES buffer to a final density of 4 × 10^8^ cells·mL^−1^ for optical aggregometry, flow cytometry and ROS release assays, and 8 × 10^8^ cells·mL^−1^ for immunoblot analysis.

### Aggregation

Platelet aggregation assays were performed by optical aggregometry stimulated with the thromboxane A_2_ receptor agonist U46619 (Cayman Chemical) or the collagen receptor glycoprotein VI agonist, cross-linked collagen-related peptide (CRP-XL) (supplied by Prof R. Farndale, University of Cambridge, UK) [30]. Platelets were stimulated in this way in the presence or absence of LPS from *E*. *coli* O111:B4 (Sigma Aldrich, UK) or from *E*. *coli* K12 (purified as described above), superoxide dismutase (SOD, Sigma Aldrich, UK), catalase (Sigma Aldrich, UK), inhibitor of PI3K- LY294002 (Selleckchem, UK), inhibitor of PI3K-δ- Cal (Selleckchem, UK), Akt inhibitor IV (Calbiochem, UK), inhibitor of MEK- Cobimetinib (Selleckchem, UK), indomethacin (Sigma Aldrich, UK), the TXA_2_ receptor antagonist- GR32191 (Sigma Aldrich, UK), the TLR4 antagonist- LPS RS ultrapure (InvivoGen, UK) or vehicle used to dissolve each of the above (containing 0.01% (v/v) dimethyl sulphoxide, which did not affect platelet function, or distilled water). Aggregation assays were performed with 250μL of washed platelet suspension after incubation for 3 minutes with respective inhibitors followed by stimulation with agonist or agonist plus LPS from *Escherichia coli*- K12 or from *Escherichia coli***-** O111:B4 for 5 minutes. Aggregation was monitored using Chrono-Log Model 700/ Optical-Lumi Aggregometer.

### Immunobloting assay

SDS-PAGE and immunoblotting were performed using standard protocols as described previously [31]. Rabbit anti-human 14-3-3ζ (Santa Cruz Biotechnology, USA) was used to detect 14-3-3ζ to ensure equivalent levels of protein loading on immunoblots. The phospho-specific antibodies against various signalling proteins (Akt, ERK1/2 and PLA_2_) were obtained from Cell signalling technology, USA (catologue numbers: 9271, 4370 and 2831 respectively). The secondary antibodies for immunoblotting; Cy5 goat anti-rabbit IgG and Cy3 goat anti-mouse IgG antibodies were obtained from Invitrogen, UK. Blots were visualised using a Typhoon FLA 9500 fluorimager (GE healthcare, UK). Image Quant TL software (GE Healthcare) was used for the fluorescence visualisation and analysis of protein bands.

### Alpha granule secretion and fibrinogen binding

Flow cytometric assays were performed in 96-well plates. U46619 or CRP-XL-stimulated fibrinogen binding and P-selectin exposure were measured in PRP using FITC labelled rabbit anti-human fibrinogen antibodies (Dako UK Ltd) and PE/Cy5 labelled mouse anti-human anti-CD62P antibody (BD Biosciences, UK) respectively in the presence or absence of different antagonists or inhibitors for 3 minutes prior to activation (e.g. superoxide dismutase- SOD, catalase, inhibitor of PI3-Kinase- LY294002, inhibitor of PI3K-δ (Cal), Akt inhibitor IV, inhibitor of MEK (cobimetinib), Indomethacin, TXA_2_ antagonist (GR32191) or TLR4 antagonist (LPS RS ultrapure) or vehicle (containing 0.01% dimethyl sulphoxide or distilled water)). Platelets were stimulated with U46619 or CRP in the presence or absence of LPS from *Escherichia coli***-** O111:B4 or from *Escherichia coli*- K12 for 5 minutes at room temperature and then fixed in 0.2% (v/v) formyl saline prior to analysis by BD Accuri flow cytometry (BD Biosciences, Oxford, UK) equipped with a 488 nm wavelength argon laser, 510–540 nm band pass filter. Data were acquired from 10000 cells and recorded as percentage of cells positive or median fluorescence intensity (MFI).

### Platelet ROS-production measured by flow cytometry

Production of ROS by platelets was measured by flow cytometry following the loading of platelets with the fluorescent dye, 2^’^,7^’^-dichlorofluorescin diacetate (DCFH-DA) (Sigma Aldrich, UK). DCFH is membrane-impermeable and rapidly oxidizes into the highly fluorescent 2´,7`-dichlorofluorescin (DCF) in the presence of intracellular hydrogen peroxide (H_2_O_2_), peroxynitrite (ONOO-), peroxides and hydroxyl radicals (·OH) [32]. Briefly, 50μL of washed platelets (4 x 10^8^/ml) were preincubated with 10μM DCFH-DA in the presence or absence of inhibitors (e.g. superoxide dismutase, catalase, inhibitor of PI3-Kinase (LY294002), inhibitor of PI3K (Cal), Akt inhibitor IV, inhibitor of MEK- (cobimetinib), indomethacin, TXA_2_ receptor antagonist (GR32191) or TLR4 antagonist (LPS RS ultrapure), or vehicle (containing 0.01% (v/v) dimethyl sulphoxide) for 10 minutes at 37°C in the dark in a 24 well plate before stimulation with U46619 or CRP-XL, together with LPS from *Escherichia coli***-** O111:B4 or LPS from *Escherichia coli*- K12 for 10 minutes. The samples were then fixed with 200μL of sodium azide (0.1% w/v) at room temperature for 10 minutes in the dark. Samples were analysed by BD Accuri flow cytometry (BD Biosciences, Oxford, UK) equipped with a 488 nm wavelength argon laser, 510–540 nm band pass filter. Data were acquired from 10000 cells and recorded as percentage of cells positive or median fluorescence intensity (MFI).

### Statistical analysis

Data are presented as mean ± standard deviation of the mean (SEM). Statistical analyses were performed using PRISM 5 GRAPHPAD software (GraphPad Software Inc, La Jolla, CA, USA). Data were analysed to confirm normal distribution and compared using a Student’s t-Test or One-way ANOVA- and Bonferroni post-test analysis as appropriate.

## Results

### LPS *in vitro* potentiates activation of stimulated platelets

**Fig 1:**
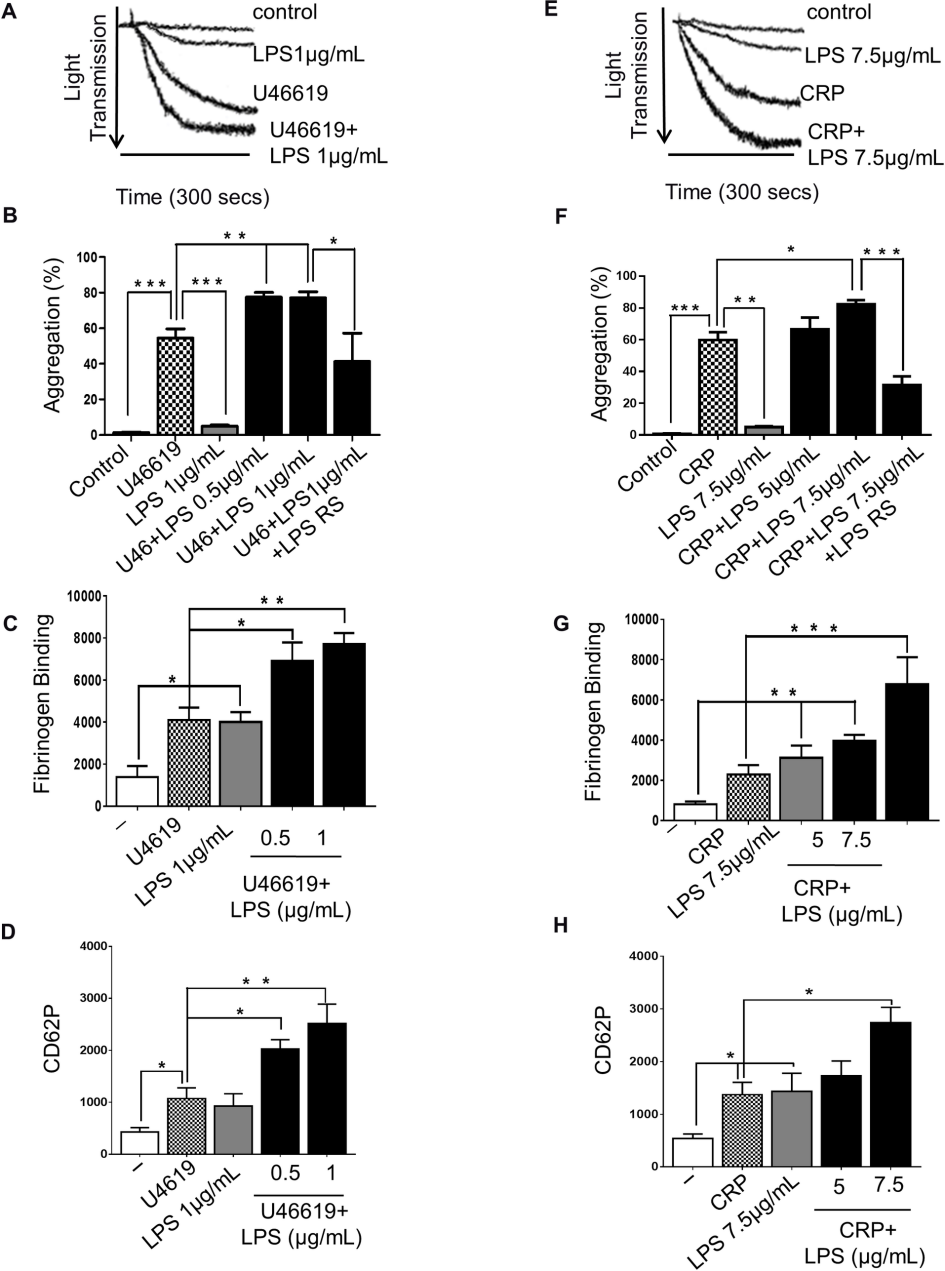
LPS potenciates U46619-stimulated platelet activation. Human-washed platelet aggregation was performed by optical aggregometry following stimulation with U46619 (0.25μM) or CRP-XL (0.25μg/ml) in the presence or absence of LPS from *E*. *coli* O111:B4 (0.5, 1, 5 or 7.5μg/mL) or LPS RS (1 or 7.5 μg/mL) (A, B, E, F). The effect of U46619 or CRP-XL and LPS- on fibrinogen binding and P-selectin exposure was measured in PRP by flow cytometry (C, D, G, H). Cumulative data represent mean values ± SEM (n = 4). (Anova-Bonferroni test, * P≤ 0.05; ** P≤ 0.01; *** P≤ 0.001).

The effect of LPS on platelet function remains uncertain, although previous work has shown that different preparations of LPS *in vitro* induce platelet aggregation [7, 33]. To analyse the effects of LPS on platelet aggregation, washed human platelets were incubated with LPS from *E*. *coli* O111:B4 or alternatively purified from *E*. *coli* K12 (0.5, 1, 5 or 7.5µg/mL) in the presence or absence of the thromboxane receptor agonist U46619 (0.25μM) or the GPVI collagen receptor agonist CRP-XL (0.25μg/mL) and analysed for 5 min in an optical aggregometer. Both sources of LPS increased significantly the aggregation of platelets stimulated with U46619 (Fig 1A and 1B (LPS: *E*. *coli* O111:B4); S2A Fig (LPS: *E*. *coli* K12)) and CRP-XL Fig 1E and 1F (LPS: *E*. *coli* O111:B4); S2E Fig (LPS: *E*. *coli* K12)) by 42% and 21% respectively. To confirm that the effects of LPS on platelets were stimulated by binding to TLR4, LPS from *Rhodobacter sphaeroides* (LPS-RS) (1μg/mL), a TLR4 antagonist, was used. LPS-RS prevented the increase in aggregation stimulated by LPS from *E*.*coli* O111:B4 in platelets stimulated with U46619 (Fig 1B) or CRP-XL (Fig 1F).

Platelet inside-out signalling leads to a conformational change in integrin αIIbβ3 converting it from a low to high affinity state, thereby allowing activation dependent binding of its ligands fibrinogen and Von Willebrand Fator (VWF) [34]. This is also associated with the secretion of α-granules which contain adhesion proteins such as fibrinogen, VWF, coagulation and fibrinolytic factors [35]. To analyse the effects of LPS on the modulation of integrin αIIbβ3 affinity and α-granule secretion, fibrinogen binding and P-selectin exposure on the surface of platelets were measured by flow cytometry. Although both LPS from *E*. *coli* O111:B4 or the alternative LPS from *E*. *coli* K12 (1 and 7.5 μg/mL) caused a slight decrease in optical density of platelet suspensions (5%), LPS from *E*. *coli* O111:B4 raised approximately two-fold the level of fibrinogen binding and P-selectin exposure on unstimulated platelets. Following stimulation with U46619 (Fig 1C and 1D (LPS: *E*. *coli* O111:B4)); S2B and S2C Fig (LPS: *E*. *coli* K12)) or CRP-XL (Fig 1G and 1H (LPS: *E*. *coli* O111:B4); S2F and S2G Fig (LPS: *E*. *coli* K12)), both source of LPS were able to significantly increase fibrinogen binding (LPS from *E*. *coli* O111:B4: 70% and 200% respectively with U46619 and CRP-XL; LPS from *E*. *coli* K12: 170% and 145% respectively with U46619 and CRP-XL) and P-selectin exposure (LPS from *E*. *coli* O111:B4: 130% and 100% respectively with U46619 and CRP-XL; LPS from *E*. *coli* K12: 170% and 120% respectively with U46619 and CRP-XL).

### LPS potentiates aggregation through an increase of platelet-ROS production

**Fig 2:**
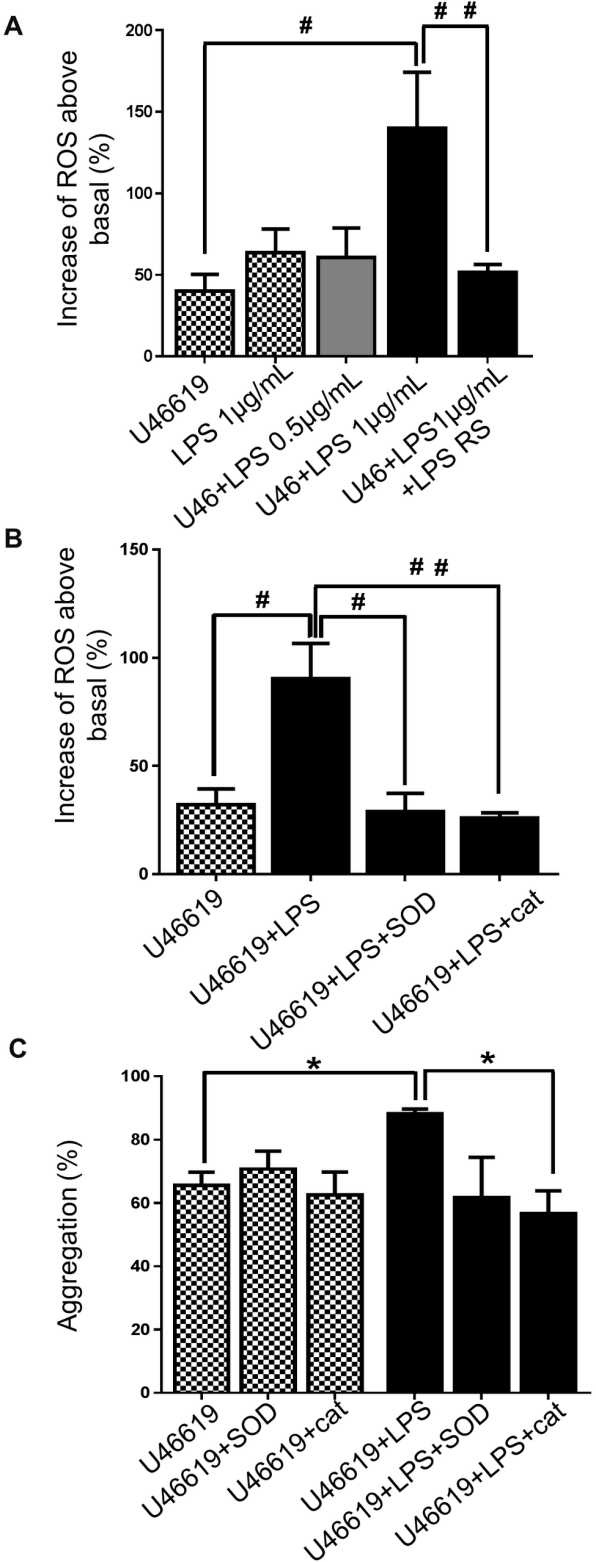
ROS release induced by LPS promotes potentiation of aggregation. Washed platelets (4 x 10^8^/ml) were pre-incubated with 10μM DCFH-DA in the presence or absence of vehicle, LPS from *E*. *coli* O111:B4 (0.5 or 1μg/mL) or LPS RS (1μg/mL), superoxide dismutase- SOD (30U/mL) or catalase (300U/mL) before being activated with U46619 (0.25μM). Samples were analysed by flow cytometry and the levels of ROS released were detected and expressed as % increase above levels detected in unstimulated platelets (A and B). Human-washed platelet aggregation was performed by optical aggregometry following stimulation with U46619 (0.25μM) in the presence or absence of LPS from *E*. *coli* (1μg/mL) after 3 min of incubation with superoxide dismutase- SOD (30U/mL) or catalase (300U/mL) (C). Cumulative data represent mean values ± SEM (n = 4). (Anova-Bonferroni test, * P≤ 0.05; Test t student ^#^ P≤ 0.01; ^# #^ P≤ 0.01).

Platelets are widely recognised to contribute to inflammation and themselves can generate endogenous inflammatory mediators and ROS [14, 15]. To investigate the capacity of LPS to induce platelet ROS-generation by TLR4 binding and to explore the cell signalling that may control this, ROS production was measured in stimulated and unstimulated platelets by flow cytometry using the DCFH-DA fluorescent ROS probe (10μM). Stimulation of platelets with U46619 was associated with an increase in platelet- derived ROS. Both sources of LPS (1 and 7.5 μg/mL) promoted approximately three-fold increase of ROS release in platelets stimulated with U46619 (0.25μM) (Fig 2A and 2B (LPS: *E*. *coli* O111:B4); S2D Fig (LPS: *E*. *coli* K12). LPS from *E*. *coli* O111:B4 and from *E*. *coli* K12 were able also to raise by approximately 70% the ROS release of unstimulated platelets (Fig 2A (LPS: *E*. *coli* O111:B4 S2D and S2H Fig (LPS: *E*. *coli* K12)). Incubation with TLR4 antagonist- LPS RS ultrapure decreased substantially the production of ROS raised by LPS from *E*. *coli* O111:B4 in platelets stimulated with U46619 (0.25μM, reduction of 60%) (Fig 2A), suggesting that LPS potentiates ROS generation in platelets through TLR4 binding.

Platelet activation can be modulated by ROS derived from sources including stimulated platelets themselves, such as superoxide anions (O^-2^) and hydrogen peroxide (H_2_O_2_) [36]. To analyse whether LPS-potentiated platelet aggregation was associated with the modulation of O^-2^ and H_2_O_2_ production, platelets were incubated with superoxide dismutase- SOD (30U/mL) or catalase (300U/mL) (inhibitors of O^-2^ and H_2_O_2_ respectively), prior to being stimulated with U46619 (0.25μM). Both SOD and catalase were able to reduce the LPS- *E*. *coli* O111:B4 -induced increases in aggregation and ROS generation to a similar level to that seen in the absence of LPS (Fig 2B and 2C). These analyses indicate that H_2_O_2_ and O^-2^ are likely to contribute to the potentiation of aggregation mediated by LPS *in vitro*.

### LPS potentiates platelets responses via PI3K/Akt signalling

**Fig 3:**
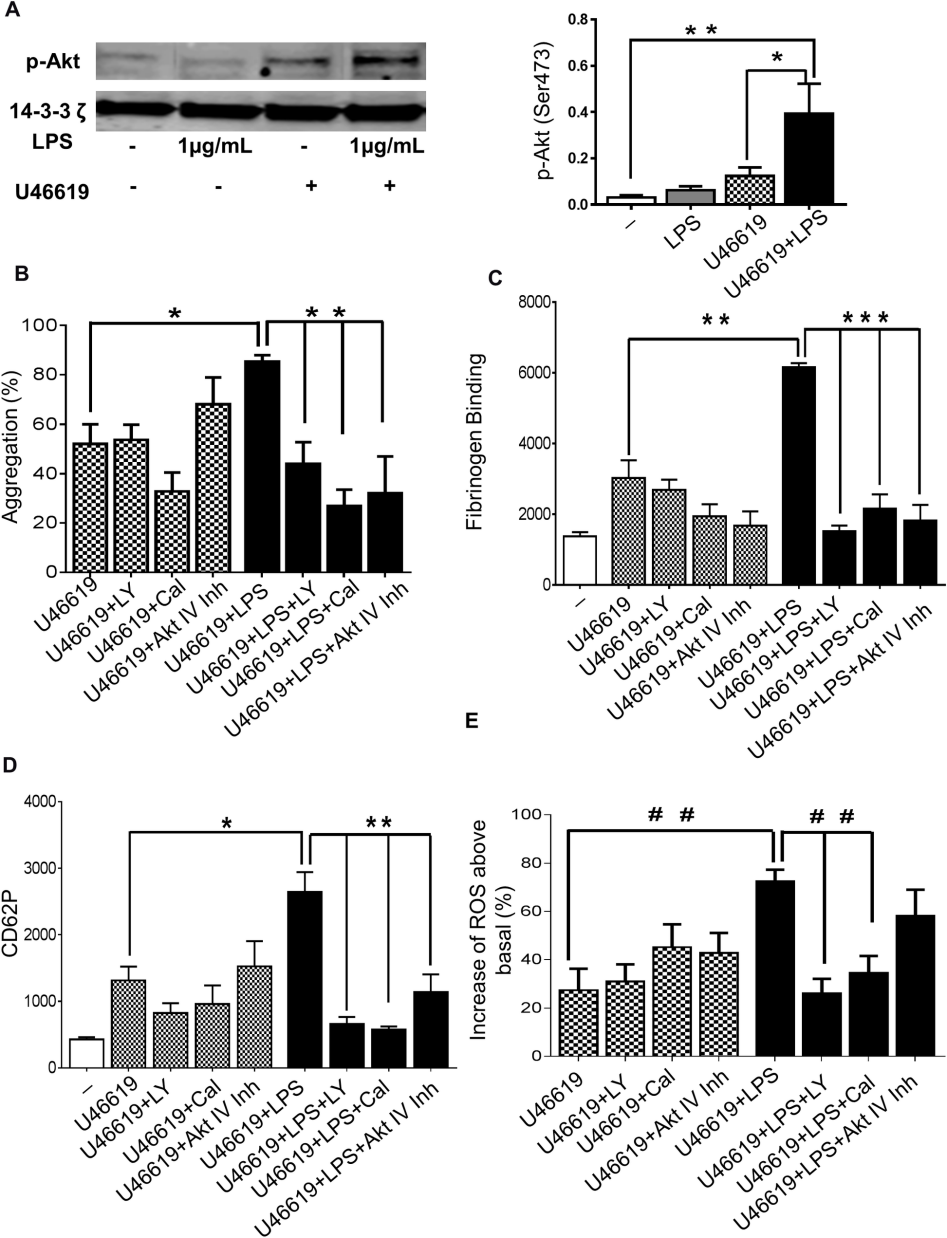
Exposure of platelets to LPS potentiates PI3K/Akt signalling. Washed human platelets with or without treatment with U46619 (0.25μM) in the presence or absence of LPS from *E*. *coli* O111:B4 (1μg/mL) were analysed by immunoblotting using antiphospho-Akt (Ser473) antibody. Total levels of 14-3-3 ζ were measured on each sample as a loading control (A). Human-washed platelet aggregation was performed by optical aggregometry activated with U46619 (0.25μM) in the presence or absence of LPS from *E*. *coli* O111:B4 (1μg/mL) after 3 min of incubation with LY294002 (20μM), Cal (60μM) or Akt inhibitor IV (5μM) (B). The effect of U46619 and LPS- induced fibrinogen binding and P-selectin exposure after incubation with LY294002 (20μM), Cal (60μM) or Akt inhibitor IV (5μM) were measure in PRP by flow cytometry (C and D). Washed platelets (4 x 10^8^/ml) were pre-incubated with 10μM DCFH-DA in the presence or absence of LY294002 (20μM), Cal (60μM) or Akt inhibitor IV (5μM) before being activated with U46619 (0.25μM) in the presence or absence of LPS from *E*. *coli* O111:B4 (1μg/mL) and ROS levels were analysed by flow cytometry (E). Cumulative data represent mean values ± SEM (n = 4). (Anova-Bonferroni test, * P≤ 0.05; ** P≤ 0.01; *** P≤ 0.001; Test t student ^# #^ P≤ 0.01).

Phosphoinositide 3-kinase (PI3K)/Akt-dependent signalling is involved in activation of integrin αIIbβ3 and regulates platelet secretion [23]. Previous studies have shown the involvement of the PI3K-Akt pathway in LPS-mediated effects in various cells [25, 26]. To assess the effect of LPS *in vitro* on PI3K-Akt dependent signalling in platelets, we employed western blotting to measure Akt phosphorylation on Ser 473. LPS (1μg/mL) raised by three-fold the phosphorylation of Akt in platelets stimulated with U46619 (0.25μM) (Fig 3A). Platelets were incubated with a pan- PI3K isoform inhibitor LY294002 (20μM), an inhibitor of PI3K-δ- Cal (60μM) or the Akt inhibitor IV (5μM) and platelet function was measured. The increase of aggregation induced by LPS in platelets stimulated with U46619 (0.25μM, (Fig 3B) or CRP-XL (0.25μg/mL, (S3A Fig)) was reduced significantly by the three inhibitors (approximately 50% inhibition for LY294002 and 70% for Cal or Akt inhibitor). Consistent with their effects on aggregation, the three inhibitors decreased substantially fibrinogen binding and P-selectin exposure of platelets stimulated with U46619 (0.25μM) and LPS (1μg/mL) (Fig 3C and 3D). LY294002 and Cal reduced the increase of ROS produced by LPS treatment to the level observed in the absence of LPS in platelets stimulated with U46619 (Fig 3E), although CRP-XL-stimulated ROS production was not inhibited by these inhibitors (S3B Fig). Together these data suggest that the PI3K/Akt pathway cooperates with LPS signalling which promotes enhanced platelet activation through the release and actions of ROS.

### Akt mediates LPS pathway through ERK1/2 and PLA_2_- phosphorylation

**Fig 4:**
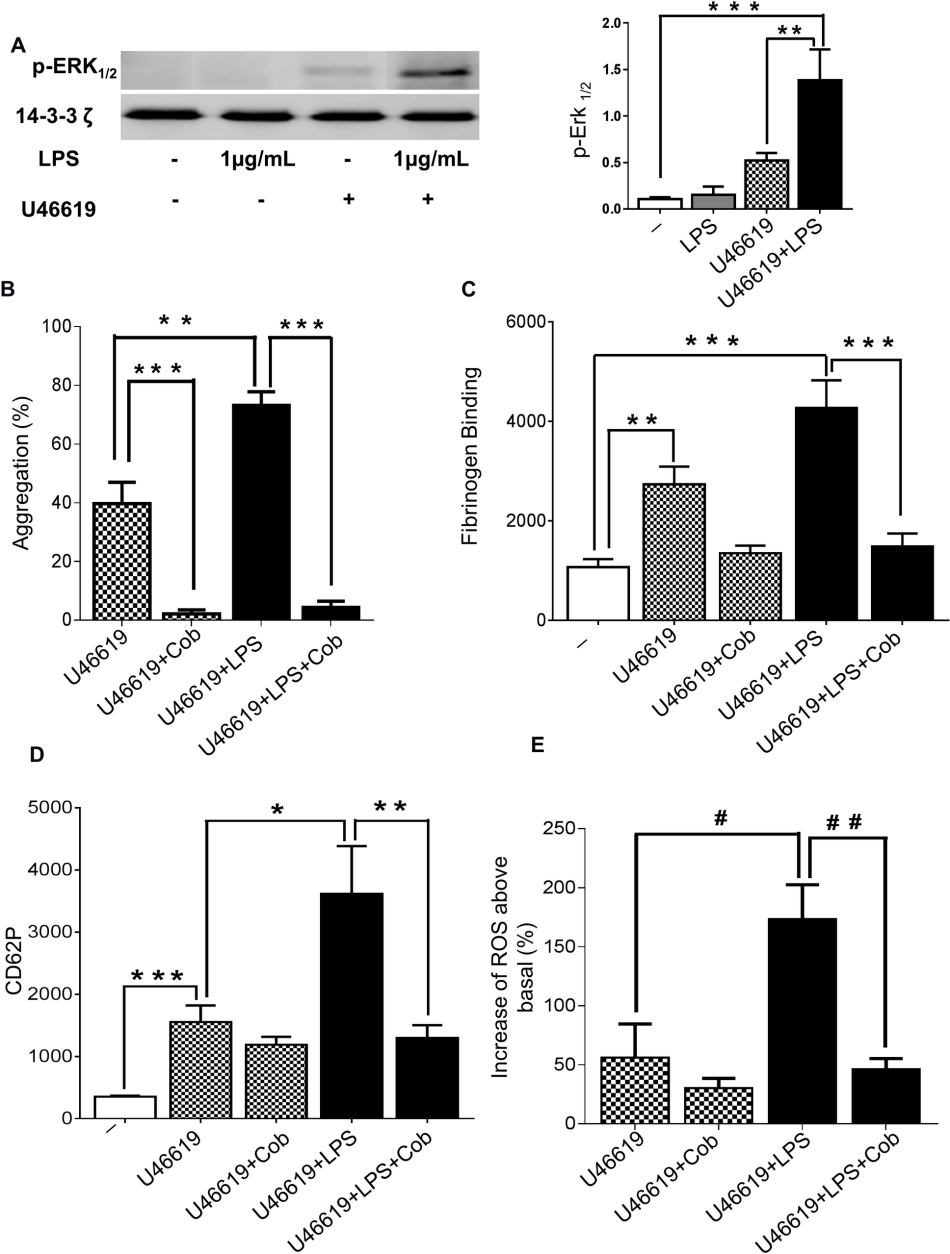
The role of ERK1/2 in LPS-mediated platelet potentiation. Washed human platelets stimulated or not with U46619 (0.25μM) in the presence or absence of LPS from *E*. *coli* O111:B4 (1μg/mL) were analysed by immunoblotting using antiphospho- ERK1/2 antibody. Total levels of 14-3-3 ζ were measured on each sample as a loading control (A). Human-washed platelet aggregation was performed by optical aggregometry following stimulation with U46619 (0.25μM) in the presence or absence of LPS from *E*. *coli* O111:B4 (1μg/mL) after 3 min of incubation with Cobimetinib (100μM) before activation with U46619 (0.25μM) (B). The effect of U46619 (0.25μM) and LPS- induced fibrinogen binding and P-selectin exposure after incubation with Cobimetinib (100μM) were measured in PRP by flow cytometry (C and D). Washed platelets (4 x 10^8^/mL) were pre-incubated with 10μM DCFH-DA in the presence or absence of Cobimetinib (100μM) before being activated with U46619 (0.25μM) in the presence or absence of LPS from *E*. *coli* O111:B4 (1μg/mL) and ROS levels were analysed by flow cytometry (E). Cumulative data represent mean values ± SEM (n = 4). (Anova-Bonferroni test, * P≤ 0.05; ** P≤ 0.01; *** P≤ 0.001; Test t student ^#^ P≤ 0.05; ^# #^P≤ 0.01).

ERK activation plays an essential role in mediating platelet granule release by amplifying platelet responses and promoting a second wave of platelet aggregation [24]. It has previously been suggested that cell signalling promoted through the binding of LPS with TLR4 involves ERK1/2 phosphorylation and activation [31]. The ability of LPS (1μg/mL) to modulate ERK1/2 phosphorylation was therefore tested using immunoblot analysis. In stimulated platelets (U46619- 0.25μM), ERK1/2 phosphorylation was increased by a factor of three in the presence of LPS (1μg/mL) (Fig 4A). The inhibitor of ERK1/2, Cobimetinib (100μM) reduced dramatically (approximately 95%) the aggregation of platelets induced by U46619 (Fig 4B) or CRP-XL (S4A Fig) in the presence or absence of LPS. Additionally, Cobimetinib did not significantly change fibrinogen binding and P-selectin exposure in U46619-stimulated platelets but reduced by 65% both functions in platelets stimulated in the presence of LPS (Fig 4C and 4D). The inhibitor of ERK1/2 was also able to decrease by 75% the rise of ROS production by LPS in platelets stimulated with U46619 (Fig 4E) and CRP (S4B Fig).

**Fig 5:**
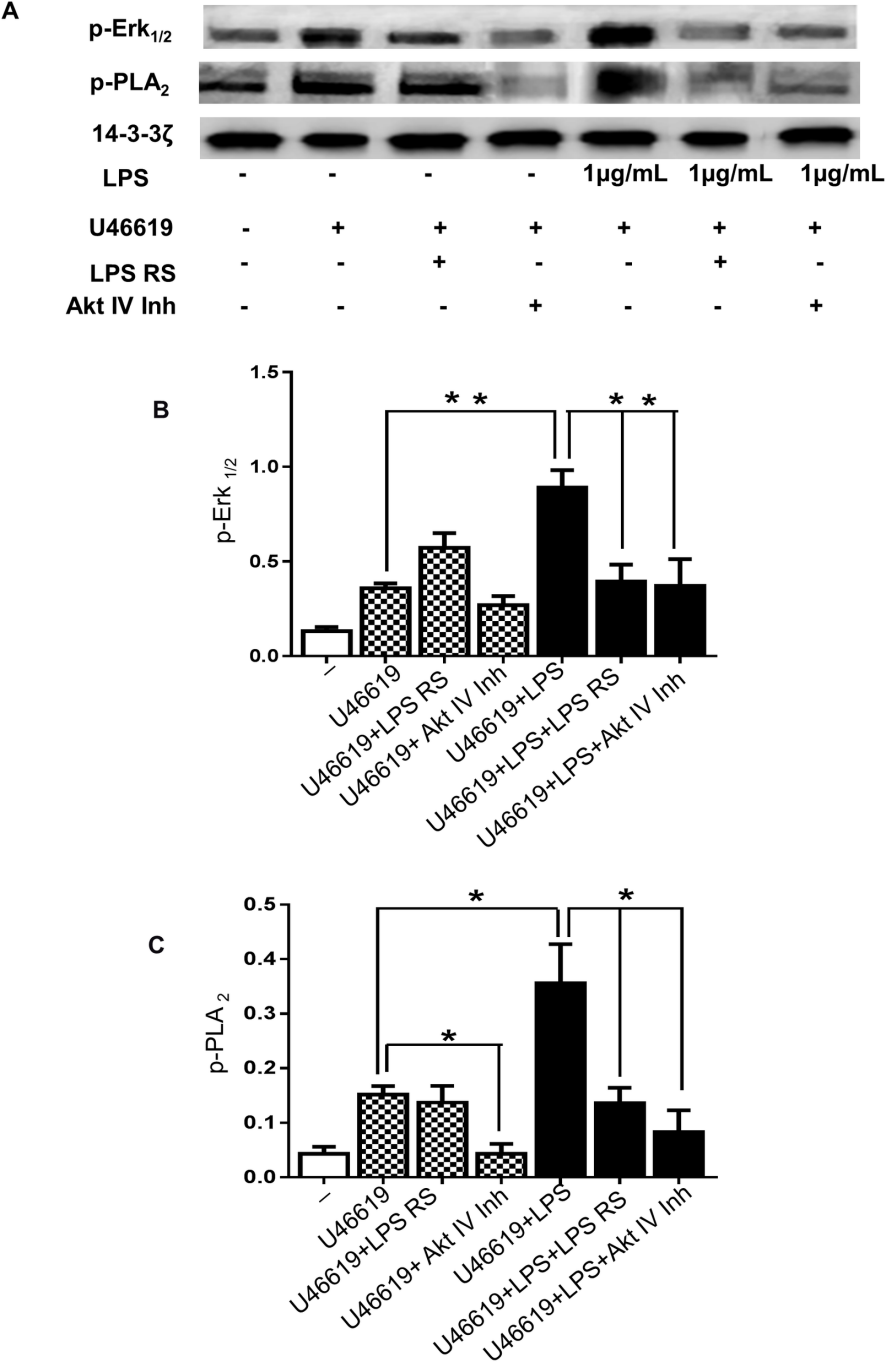
LPS stimulates ERK1/2 and PLA_2_- phosphorylation via TLR4 and Akt. Washed human platelets were incubated for 3 minutes with LPS RS (1μg/mL) or Akt inhibitor IV (5μM) before activation with U46619 (0.25μM) in the presence or absence of vehicle or LPS from *E*. *coli* (1μg/mL) and were analysed by immunoblotting using antiphospho- ERK1/2 (A and B) and antiphospho-PLA_2_ (A and C) antibodies. Total levels of 14-3-3ζ were measured on each sample as a loading control. Cumulative data represent mean values ± SEM (n = 4). (Anova-Bonferroni test, * P≤ 0.05; ** P≤ 0.01).

Previous research has shown that phosphorylation of ERK1/2 is associated with PLA_2_ activity [37] and in platelets this link is poorly understood. Given the significant effect of ERK1/2 on LPS signalling in platelet function, PLA_2_ activation was explored. Through immunoblotting we verified that platelets stimulated with U46619 (0.25μM) and LPS (1μg/mL) exhibited an increase of PLA_2_ phosphorylation of approximately 2.3-fold compared with platelets in the absence of LPS (Fig 5A and 5C). The TLR4 antagonist did not have any effect on PLA_2_ activation in platelets stimulated with U46619 but this antagonist was able to decrease PLA_2_ phosphorylation of platelets stimulated with U46619 and LPS to levels observed in the absence of LPS (Fig 5A and 5C). Indeed, in platelets stimulated with U46619 plus LPS, TLR4 antagonist reduced ERK1/2 phosphorylation to the levels observed in the absence of LPS (Fig 5A and 5B). These data suggest that ERK1/2 and PLA_2_ contribute to platelet signalling stimulated by LPS.

In order to analyse the involvement of Akt on ERK1/2 and PLA_2_ in LPS treated platelets, immunoblotting was performed with platelets stimulated by U46619 (0.25μM) in the presence or absence of Akt inhibitor IV (5μM). ERK1/2 and PLA_2_ phosphorylation induced by LPS were decreased to levels observed in absence of LPS by Akt inhibitor IV (Fig 5A–5C). These data indicate the potential effect of Akt in mediating platelet signalling through ERK1/2 and PLA_2_ activation.

### LPS signalling is associated with TXA_2_ production

**Fig 6:**
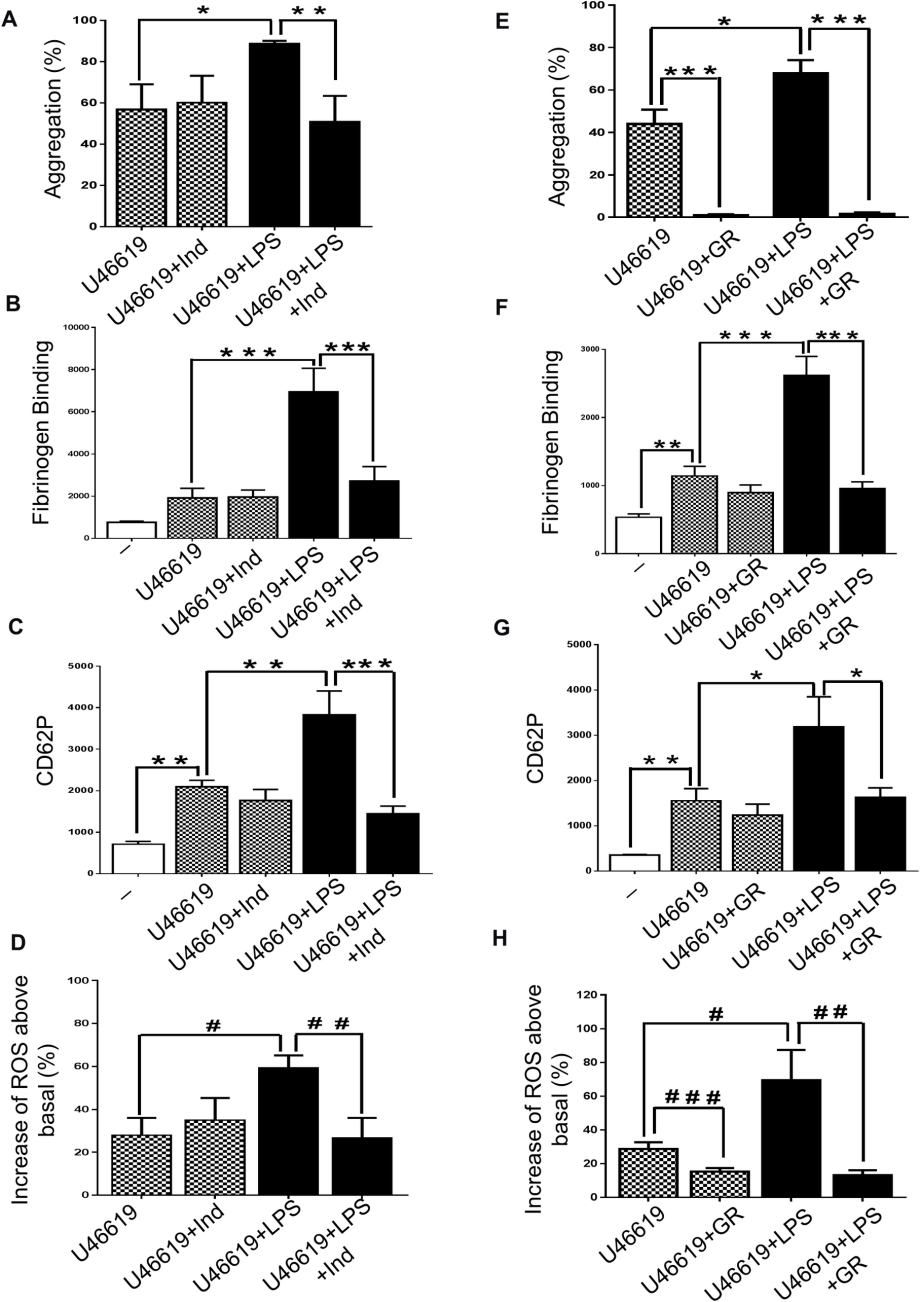
LPS- mediated platelet activation is associated with TXA_2_ production. Human-washed platelet aggregation was performed by optical aggregometry following stimulation with U46619 (0.25μM) in the presence or absence of LPS from *E*. *coli* O111:B4 (1g/mL) after 3 min of incubation with indomethacin (10μM) or GR32191 (100ng) (A and E). The effect of U46619 (0.25μM) and LPS- induced fibrinogen binding and P-selectin exposure after incubation with indomethacin (10μM) or GR32191 (100ng) were measure in PRP by flow cytometry (B, C, F and G). Washed platelets (4 x 10^8^/mL) were pre-incubated with 10μM DCFHDA in the presence or absence of Indomethacin (10μM) or GR32191 (100ng) before being activated with U46619 (0.25μM) in the presence or absence of LPS from *E*. *coli* O111:B4 (1μg/mL) and ROS levels were analysed by flow cytometry (D and H). Cumulative data represent mean values ± SEM (n = 4). (Anova-Bonferroni test, * P≤ 0.05; ** P≤ 0.01; *** P≤ 0.001; Test t student ^#^ P≤ 0.05; ^# #^ P≤ 0.01; ^# # #^ P≤ 0.001).

Thromboxane A_2_ (TXA_2_) has been suggested to contribute in the development of sepsis [38, 39] and PLA_2_ activation may be expected to result in TXA_2_ production [40]. To determine whether TXA_2_ is a downstream molecule involved in effects of TLR4 signalling in platelets, the cyclooxygenase inhibitor indomethacin (10μM) was incubated with washed platelets before activation with U46619 (0.25μM) or CRP-XL (0.25μg/mL). Indomethacin decreased significantly the LPS induced increase in platelet function: aggregation was reduced by 42% and 73% (in platelets stimulated with U46619 and CRP-XL respectively (Fig 6A; S5A Fig); fibrinogen binding and P-selectin exposure were reduced to levels seen in the absence of LPS (Fig 6B and 6C; S5B and S5C Fig); ROS production was decreased by 55% (with both antagonists plus LPS) (Fig 6D; S5D Fig).

Consistent with this, the TXA_2_ receptor antagonist- GR32191 (100ng) prevented the potentiation of aggregation induced by LPS in platelets stimulated with both U46619 (0.25μM, (Fig 6E)) or CRP-XL (0.25μg/ml, (S5E Fig)). The pre-incubation of platelets with GR32191 reduced fibrinogen binding and P-selectin exposure to the levels observed in the absence of LPS (in platelets stimulated with both agonists) (Fig 6F and 6G, S5F and S5G Fig). GR32191 also decreased by approximately 70% ROS production in platelets stimulated by U46619 and CRP-XL in the presence of LPS (Fig 6H; S5H Fig). Thus, these data indicate that the effects of LPS are mediated via TXA_2_.

### ROS released by LPS modulates ERK1/2 and PLA_2_- phosphorylation

**Fig 7:**
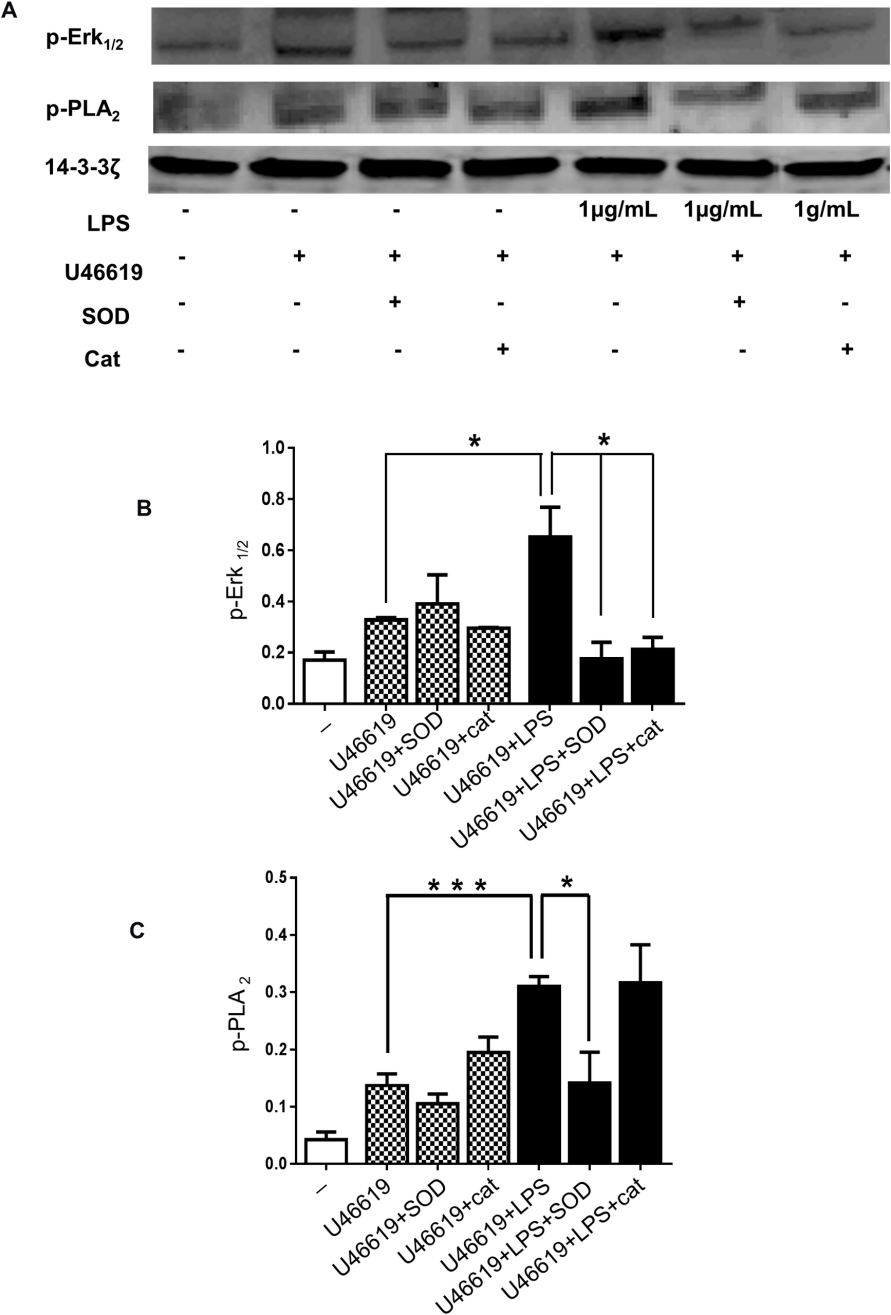
LPS induced ROS modulates ERK1/2 and PLA_2_ phosphorylation. Washed human platelets were incubated for 3 minutes with SOD (30U/mL) or catalase (300U/mL) before activation with U46619 (0.25μM) in the presence or absence of vehicle or LPS from *E*. *coli* O111:B4 (1μg/mL) and were analysed by immunoblotting using antiphospho- ERK1/2 (A and B) or antiphospho-PLA_2_ (A and C) antibodies. Total levels of 14-3-3ζ were measured on each sample as a loading control. Cumulative data represent mean values ± SEM (n = 4). (Anova-Bonferroni test, * P≤ 0.05; *** P≤ 0.001).

We have demonstrated that LPS activates ERK1/2 and PLA_2_ in platelets. To examine the role of ROS in modulating the activation of these enzymes, the levels of ERK1/2 and PLA_2_ phosphorylation in LPS-stimulated platelets were measured in the presence or absence of SOD or catalase. SOD treatment prevented LPS-induced phosphorylation of both ERK1/2 and PLA_2_ in platelets stimulated with U46619 (0.25μM), and catalase strongly inhibited ERK1/2 phosphorylation (Fig 7A–7C).

## Discussion

In the present work we have demonstrated that PI3K and Akt mediate an increase of platelet activation by LPS *in vitro* via ERK1/2/PLA_2_ pathway and our data also suggest this mechanism to involve TXA_2_ production and ROS release.

The treatment of platelets with LPS *in vitro* promoted platelet stimulation by inducing fibrinogen binding and the secretion of α-granule contents, both important markers of platelet activation. The action of LPS in platelets is a matter of some debate in the literature. The results of our study are consistent with previous studies in which LPS-induced enhancement of platelet function was observed *in vitro* [7, 8]. We found LPS to induce P-selectin secretion and activation of fibrinogen in both stimulated and unstimulated platelets, as seen in previous studies [7, 31]. We also confirmed that LPS *in vitro* enhanced aggregation of stimulated platelets, but not in resting platelets, in agreement with the work of Zhang et al (2009) and Rivadeneyra et al (2014) [7,8]. It is interesting that LPS alone did not induce substantial platelet activation, but it had an additive effect with established platelets agonists. An important limitation of existing literature in which the potential roles of LPS on platelets has been considered is that LPS derived from different bacteria are used, and the purity and quality are rarely presented. This is likely to explain some divergent results, including studies that suggest that LPS causes inhibition of platelet function following acute exposure *in vitro*. To overcome this action, in this study we employed LPS from two different strains of *E*.*coli* and TLR4-antogonising LPS. Notably, LPS purified from the two *E*. *coli* strains exerted similar effects. In addition to releasing classic inflammatory mediators, LPS promotes the generation of reactive oxygen and nitrogen species (ROS and RNS) by a range of cell types [5, 12]. Reports indicate that ROS can modulate the pathogenesis of sepsis through modulation of innate immune signalling and causing pathologic damage to cells and organs [13].

In this study we have shown that LPS promotes an increase in ROS release both in resting and in stimulated platelets. Studies have shown that O_2_^-^ reduces the threshold for platelet activation to collagen [14]. In addition, Dong et al showed that catalase *in vitro* reduces the likelihood of platelet dysfunction during LPS-induced sepsis [41]. In the present study, using SOD and catalase, we demonstrated that reactive oxygen species, such as O_2_^-^ and H_2_O_2_ do indeed contribute to enhancement of platelet function followed acute exposure to LPS.

The involvement of platelets in innate immune responses is partly due to the expression of Toll-like receptors (TLRs). Each TLR responds to a different set of ligands or PAMPs (pathogen-associated molecular patterns), lipids, lipoproteins, proteins or nucleic acids derived from bacteria, viruses and fungi [42]. Although platelets express several TLR family members [6], TLR2 and 4 have been most extensively studied because they recognise ligands in Gram-positive and negative bacteria, respectively [19] and because platelets contribute significantly to the pathophysiology of sepsis and are also involved in the high mortality levels associated with this disease [1]. After engagement, each TLR triggers its own distinctive biological response, which is specific for the PAMP recognized [6, 43]. Our study focused on the mechanisms triggered by LPS from *E*. *coli*.

Although PI3-kinase/Akt-dependent signalling has been shown to mediate some effects derived from LPS action in different cells [28, 29], it has not been established whether TLR4 signalling involves PI3-kinase/Akt activation. Here, we demonstrate that LPS *in vitro* increased Akt phosphorylation and the inhibitors of PI3K and Akt prevented the increase of platelet function and ROS production induced by LPS. Beyond the PI3K/Akt pathway, LPS stimulation also triggers the activation of small GTPases and the ERK, JNK and p38 MAPK pathways [44]. Here we found that LPS promoted ERK1/2 phosphorylation. Additionally, our findings demonstrated that TLR4 signals promote platelet stimulation and ROS release via MAPK- ERK1/2, and that is regulated by PI3K/Akt. These data are consistent with previous work in other cell types which showed PI3K/Akt and ERK1/2 to mediate a range of biological effects [21, 45, 46].

The binding of platelet integrin α_IIb_β_3_ to ligands such as fibrinogen and VWF during aggregation/adhesion contributes to platelet thrombus formation by promoting the secretion of internal granules and the stimulation of a secondary wave of aggregation, together with the formation of membrane vesicles with procoagulant activities [47]. These result in the phosphorylation of tyrosine and serine/threonine residues of several proteins including ERK, myosin light chain (MLC) and cytoplasmic phospholipase A_2_ (cPLA_2_) [48]. In this context, our data show that in stimulated platelets, LPS increases PLA_2_ activation via the actions of Akt.

During activation by various agonists, platelets undergo a cascade of events that result in the enzymatic metabolism of arachidonic acid (AA) [48]. AA is derived from the plasma membrane by the action of PLA_2_, and undergoes further metabolism by COX and TXA_2_ synthase to form eicosanoid products such as prostaglandins (PGs), thromboxane (TX), and other oxygenated derivatives [49]. One of the downstream consequences of TLR signalling is cyclooxygenase activation and the formation of pro-inflammatory cytokines. In the context of platelets a notable prostaglandin produced by COX-1 is TXA_2_ [50]. Various studies have reported the increased release of TXA_2_ in the liver [37] and kidneys [27] in endotoxemia. Here, we sought to verify if TXA_2_ is associated with the *in vitro* effects of LPS in platelets. Indeed, our results demonstrated that indomethacin, an inhibitor of COX-1 and GR32191, an antagonist of the TXA_2_ receptor decreased the effect of LPS on platelet function. Our results suggest that TXA_2_ produced by platelets is necessary for effective LPS actions. Consistent with this, Nocella et al shows that the effects of LPS are inhibited by a TXA_2_ antagonist [46]. Kassouf et al report that thrombin-induced TXB_2_ formation (a stable TXA_2_ metabolite) in platelets is suppressed by Akt inhibitor IV [51]. Here we propose that the pathway PI3K/Akt/ERK1/2/PLA_2_ leads to TXA_2_ formation following the exposure of platelets to LPS.

Gram-negative pathogens such as *E*.*coli* induce platelet hyperactivity and ROS release, which may add to the potentiation of the risk of vascular pathology. ROS can modulate LPS-TLR4 signalling at different levels and promote an increase of consecutive stimuli in immune cell [52]. Indeed, our results demonstrate that SOD was able to reduce ERK1/2 and PLA_2_ phosphorylation and catalase reduced ERK1/2 activation, showing that ROS release acts to modulate the signalling pathway following the binding of LPS to TLR4.

The involvement of PI3K, Akt and MAPK in the stimulation of platelets by agonists is established. In this work we showed that LPS *in vitro* promotes an increase of platelet function via proteins associated with the activation of platelets including Akt, ERK and PLA_2_. The effects of LPS *in vivo* are likely to be more complex, since the range of cytokines released by platelets and other cells, such as TNFα, interleukins and TGFβ may result in a systemic inflammatory state [53, 54, 55].

Disseminated intravascular coagulation associated with sepsis is present in approximately 35% of severe cases, developing to microvascular dysfunction and death [56]. Platelet activation is an essential event involved in the pathophysiological processes of the coagulation system during sepsis [57]. PLA_2_ inhibitors have gained particular attention since this enzyme is activated during inflammatory events and its expression is increased in several diseases [58] including septic shock [59]. In equine platelets and leukocytes, LPS stimulation *in vivo* increases TXA_2_ production and p38 MAPK phosphorylation to levels similar that reported in clinical endotoxaemia [60]. Additionally, a study performed *ex vivo* has reported that LPS stimulation of TLR4 potentiates platelet activation and TXA_2_ production in patients with community-acquired pneumonia [46]. The impact of increase secretion of factors from platelets and endothelial cells in response to LPS is also likely to have complex effects on homeostasis and potentially thrombosis, with some factors enhancing this, while others such as thrombomodulim, serving to inhibit coagulation [61].

In summary, our findings demonstrate that TLR4 triggered by acute exposure of platelets to LPS promotes platelet stimulation via ERK1/2 and PLA_2_ activation and that is co-ordinately regulated by PI3K/Akt, and provide evidence that this signalling pathway involves TXA_2_ production and ROS generation.

## Supporting information

S1 Fig Analysis of lipopolysaccharides by polyacrilamide gel electrophoresis. LPS standard from *E*. *coli* serotype 055:B5 and LPS from *E*. *coli* K12 were separated by 12% acrylamide gel electrophoresis and stained using Pro-Q Emerald 300 Lipopolysaccharide Gel Stain Kit.
(TIF)

S2 Fig Effect of LPS from E.coli K12 on human platelet activation. Human-washed platelet aggregation was performed by optical aggregometry following stimulation with U46619 (0.25μM) or CRP-XL (0.25μg/mL) in the presence or absence of LPS from *E*.*coli* K12 (1 or 7.5μg/mL) (A and E). The effects of U46619 (0.25μM) or CRP (0.25μg/mL) and LPS on fibrinogen binding and P-selectin exposure were measured in PRP by flow cytometry (B, C, F and G). Washed platelets (4 x 10^8^/mL) were pre-incubated with 10μM DCFHDA before being activated with U46619 (0.25μM) or CRP-XL (0.25μg/mL) in the presence or absence of LPS from *E*.*coli* K12 (1 or 7.5μg/mL) and ROS levels were analysed by flow cytometry (D and H). Cumulative data represent mean values ± SEM (n = 4). (Anova-Bonferroni test, * P≤ 0.05; ** P≤ 0.01; *** P≤ 0.001; Test t student ^#^ P≤ 0.05).
(TIF)

S3 Fig LPS potentiates activation of platelets stimulated by CRP via the PI3K/Akt pathway. Human-washed platelet aggregation was performed by optical aggregometry activated with CRP-XL (0.25μg/mL) in the presence or absence of LPS (7.5μg/mL) after 3 min of incubation with LY294002 (20μM), Cal (60μM) or Akt inhibitor IV (5μM) (A). Washed platelets (4 x 10^8^/mL) were pre-incubated with 10μM DCFH-DA in the presence or absence of LY294002 (20μM), Cal (60μM) or Akt inhibitor IV (5μM) before being activated with CRP-XL (0.25μg/mL) in the presence or absence of LPS from *E*. *coli* O111:B4 (7.5μg/mL) and ROS levels were analysed by flow cytometry. Cumulative data represent mean values ± SEM (n = 4). (Anova-Bonferroni test, * P≤ 0.05; ** P≤ 0.01; *** P≤ 0.001; Test t student ^#^ P≤ 0.05).
(TIF)

S4 Fig LPS signalling pathway requires ERK1/2 in CRP-stimulated platelets. Aggregation of human washed platelet was measured by optical aggregometry following stimulation with CRP-XL (0.25μg/mL) in the presence or absence of LPS from *E*. *coli* O111:B4 (7.5μg/mL) after 3 min of incubation with Cobimetinib (100μM) (A). Washed platelets (4 x 10^8^/mL) were pre-incubated with DCFH-DA (10μM) in the presence or absence of Cobimetinib (100μM) before being activated with CRP-XL (0.25μg/mL) in the presence or absence of LPS from *E*. *coli* O111:B4 (7.5μg/mL) and ROS levels were analysed by flow cytometry. Cumulative data represent mean values ± SEM (n = 4). (Anova-Bonferroni test, ** P≤ 0.01; *** P≤ 0.001; Test t student ^# #^ P≤ 0.01).
(TIF)

S5 Fig LPS-mediated platelet activation is associated with TXA_2_ production. Aggregation of human washed platelet was measured by optical aggregometry following stimulation with CRP-XL (0.25μg/mL) in the presence or absence of LPS from *E*. *coli* O111:B4 (7.5g/mL) after 3 min of incubation with Indomethacin (10μM) or GR32191 (100ng) (A and E). The effect of CRP-XL (0.25μg/ml) and LPS- on fibrinogen binding and P-selectin exposure after incubation with indomethacin (10μM) or GR32191 (100ng) were measure in PRP by flow cytometry (B, C, F and G). Washed platelets (4 x 10^8^/mL) were pre-incubated with DCFHDA (10μM) in the presence or absence of Indomethacin (10μM) or GR32191 (100ng) before being stimulated with CRP-XL (0.25μg/ml) in the presence or absence of LPS from *E*. *coli* O111:B4 (7.5μg/mL) and ROS levels were analysed by flow cytometry (D and H). Cumulative data represent mean values ± SEM (n = 4). (Anova-Bonferroni test, * P≤ 0.05; ** P≤ 0.01; *** P≤ 0.001; Test t student ^#^ P≤ 0.05; ^# # #^ P≤ 0.001).
(TIF)
