# Supplementary figures and images for: Lipopolysaccharide potentiates platelet responses via toll-like receptor 4-stimulated Akt-Erk-PLA2 signalling
Source: PLoS One. 2017 Nov 14;12(11):e0186981. doi: 10.1371/journal.pone.0186981 (PMC5685579; doi:10.1371/journal.pone.0186981)

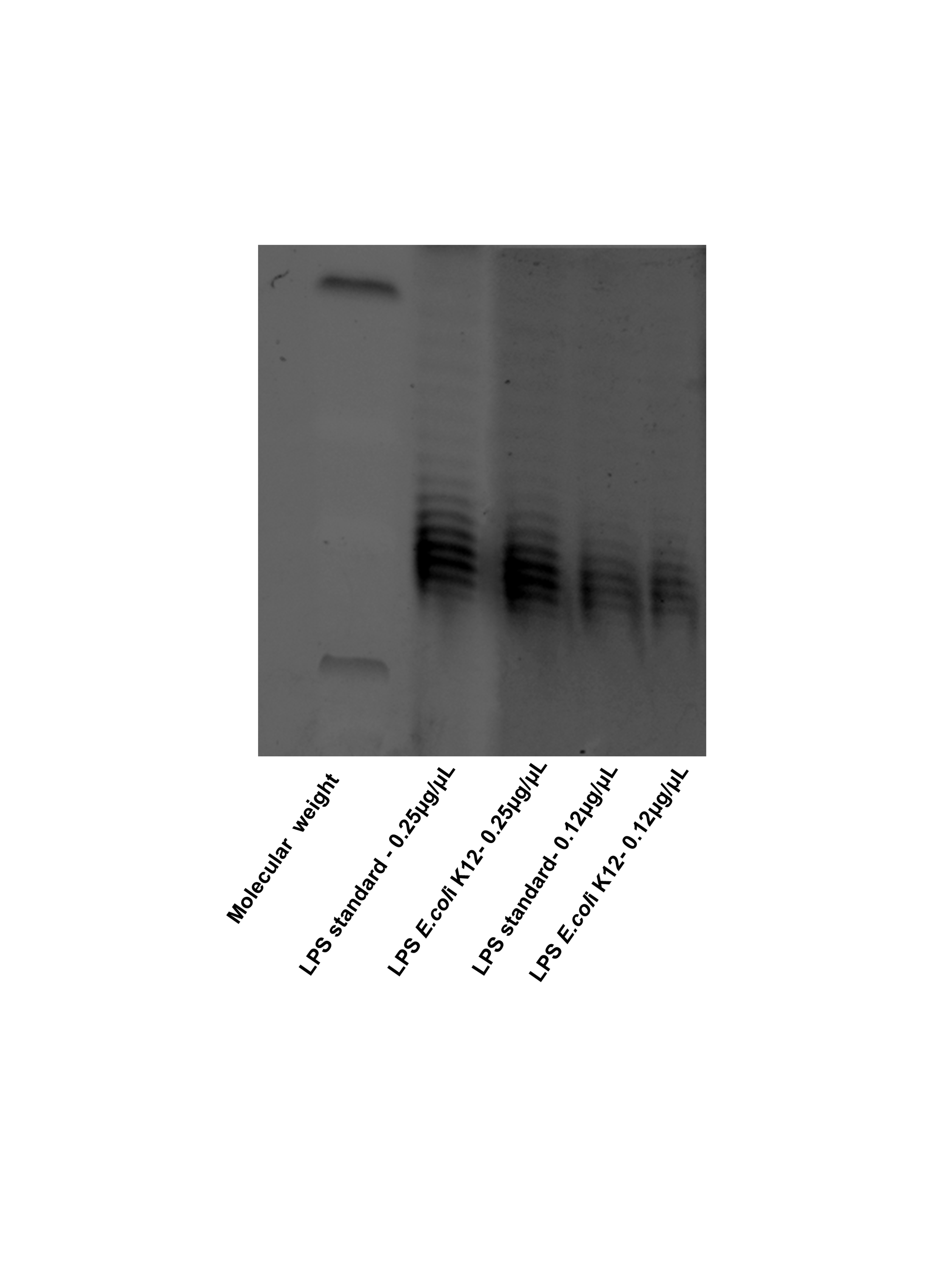

Supplement: S1 Fig — LPS standard from E. coli serotype 055:B5 and LPS from E. coli K12 were separated by 12% acrylamide gel electrophoresis and stained using Pro-Q Emerald 300 Lipopolysaccharide Gel Stain Kit. (TIF) [file pone.0186981.s001.tif]

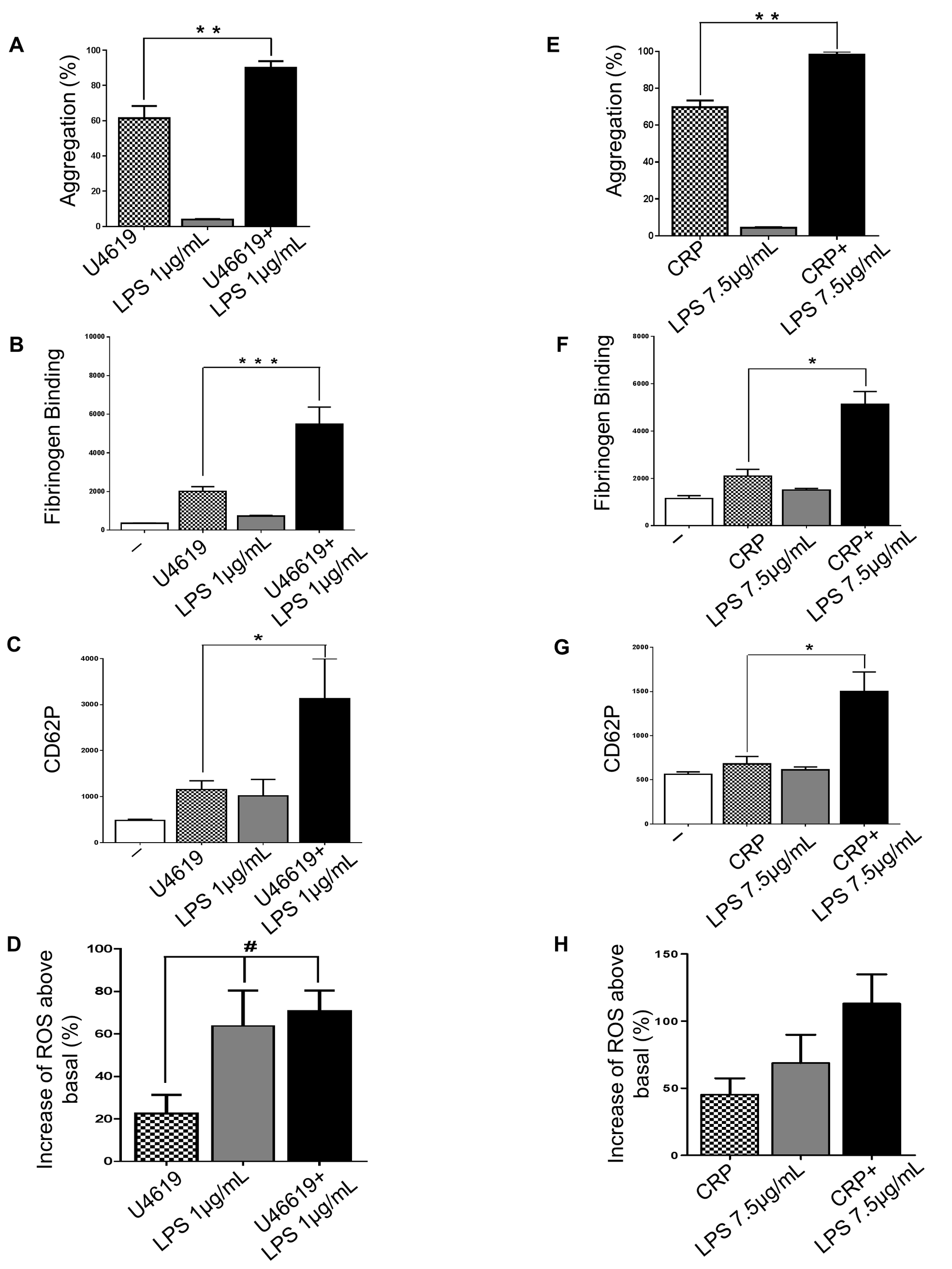

Supplement: S2 Fig — Human-washed platelet aggregation was performed by optical aggregometry following stimulation with U46619 (0.25μM) or CRP-XL (0.25μg/mL) in the presence or absence of LPS from E.coli K12 (1 or 7.5μg/mL) (A and E). The effects of U46619 (0.25μM) or CRP (0.25μg/mL) and LPS on fibrinogen binding and P-selectin exposure were measured in PRP by flow cytometry (B, C, F and G). Washed platelets (4 x 10^8^/mL) were pre-incubated with 10μM DCFHDA before being activated with U46619 (0.25μM) or CRP-XL (0.25μg/mL) in the presence or absence of LPS from E.coli K12 (1 or 7.5μg/mL) and ROS levels were analysed by flow cytometry (D and H). Cumulative data represent mean values ± SEM (n = 4). (Anova-Bonferroni test, * P≤ 0.05; ** P≤ 0.01; *** P≤ 0.001; Test t student # P≤ 0.05). (TIF) [file pone.0186981.s002.tif]

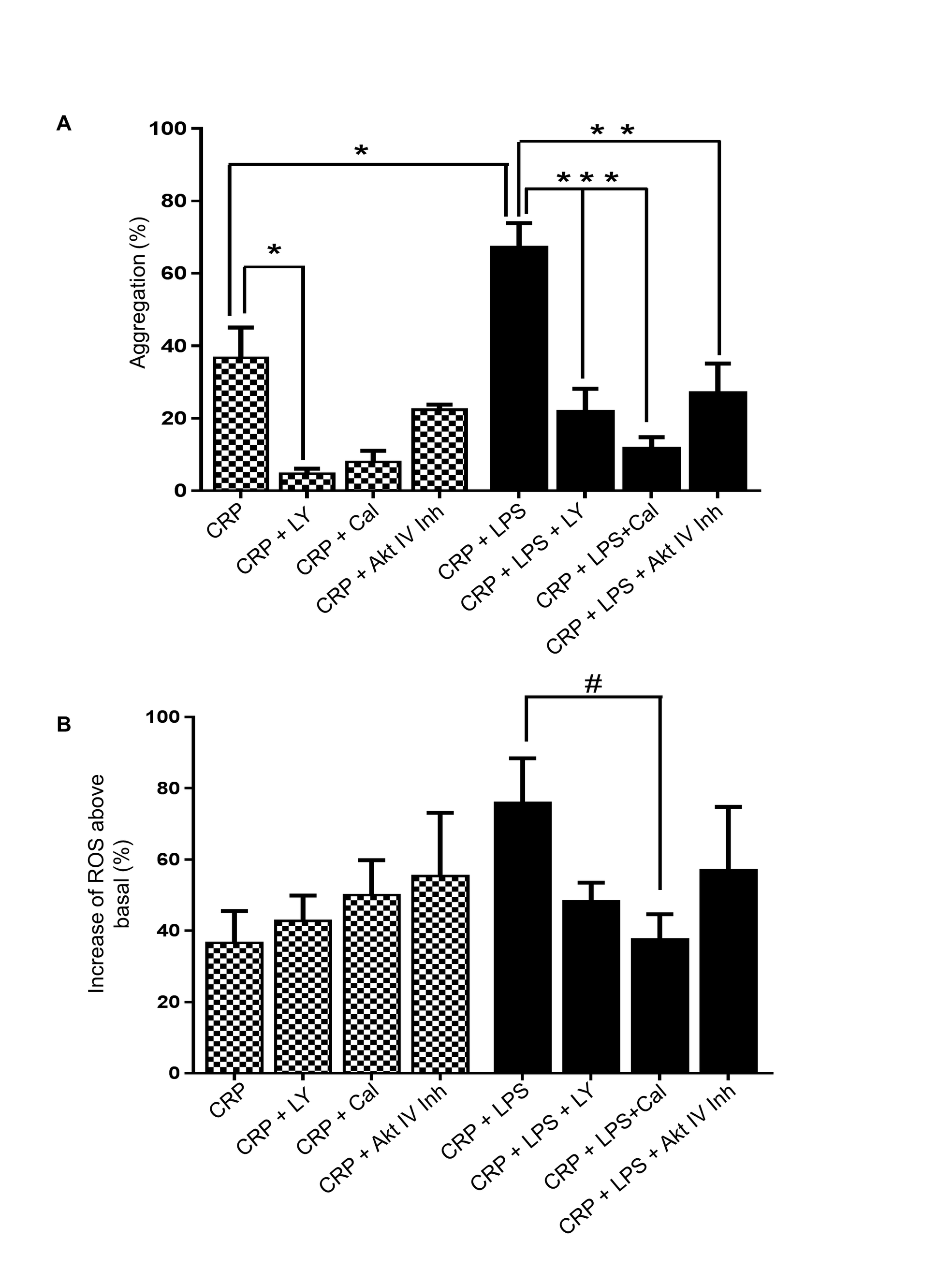

Supplement: S3 Fig — Human-washed platelet aggregation was performed by optical aggregometry activated with CRP-XL (0.25μg/mL) in the presence or absence of LPS (7.5μg/mL) after 3 min of incubation with LY294002 (20μM), Cal (60μM) or Akt inhibitor IV (5μM) (A). Washed platelets (4 x 10^8^/mL) were pre-incubated with 10μM DCFH-DA in the presence or absence of LY294002 (20μM), Cal (60μM) or Akt inhibitor IV (5μM) before being activated with CRP-XL (0.25μg/mL) in the presence or absence of LPS from E. coli O111:B4 (7.5μg/mL) and ROS levels were analysed by flow cytometry. Cumulative data represent mean values ± SEM (n = 4). (Anova-Bonferroni test, * P≤ 0.05; ** P≤ 0.01; *** P≤ 0.001; Test t student # P≤ 0.05). (TIF) [file pone.0186981.s003.tif]

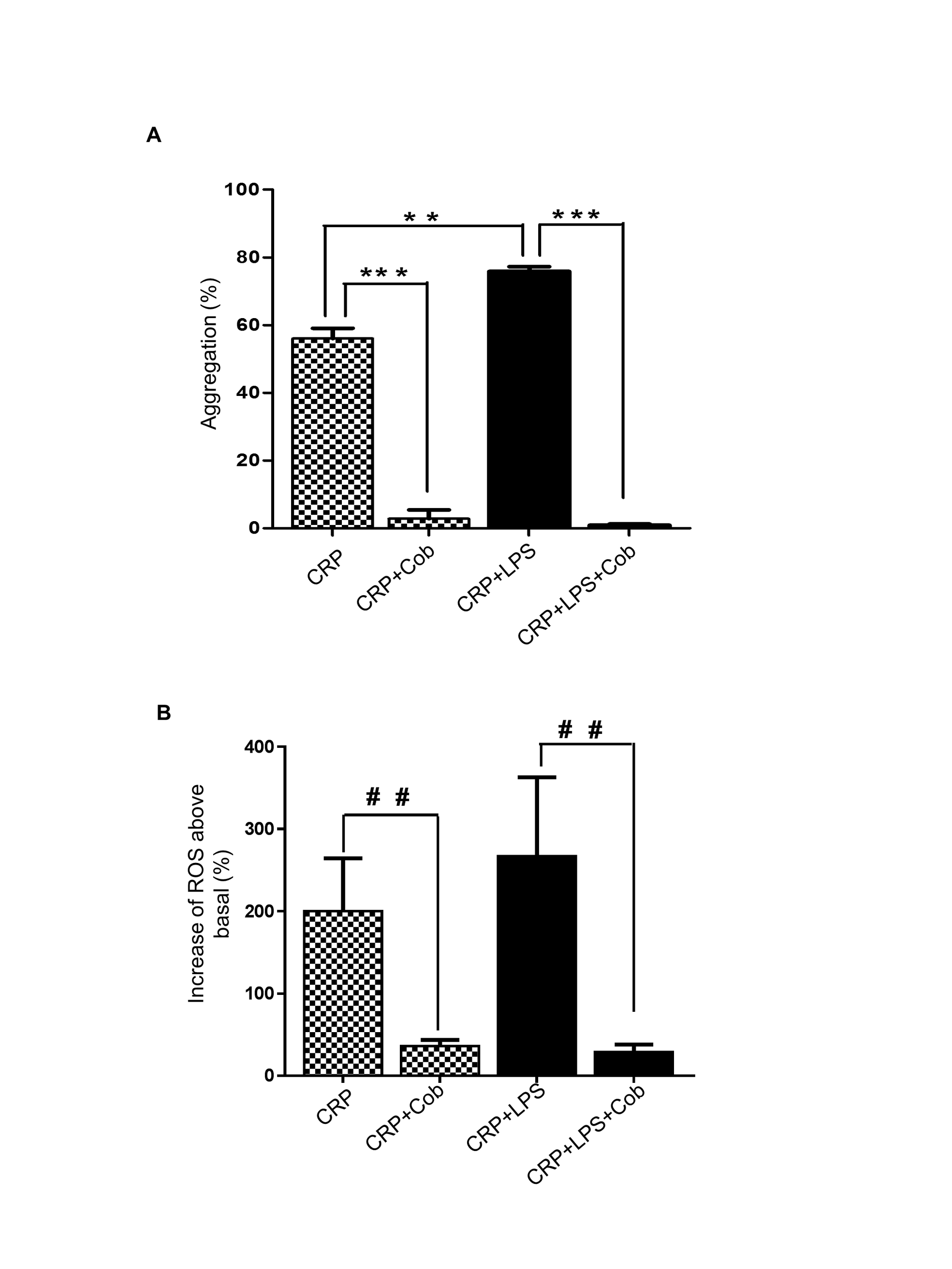

Supplement: S4 Fig — Aggregation of human washed platelet was measured by optical aggregometry following stimulation with CRP-XL (0.25μg/mL) in the presence or absence of LPS from E. coli O111:B4 (7.5μg/mL) after 3 min of incubation with Cobimetinib (100μM) (A). Washed platelets (4 x 10^8^/mL) were pre-incubated with DCFH-DA (10μM) in the presence or absence of Cobimetinib (100μM) before being activated with CRP-XL (0.25μg/mL) in the presence or absence of LPS from E. coli O111:B4 (7.5μg/mL) and ROS levels were analysed by flow cytometry. Cumulative data represent mean values ± SEM (n = 4). (Anova-Bonferroni test, ** P≤ 0.01; *** P≤ 0.001; Test t student # # P≤ 0.01). (TIF) [file pone.0186981.s004.tif]

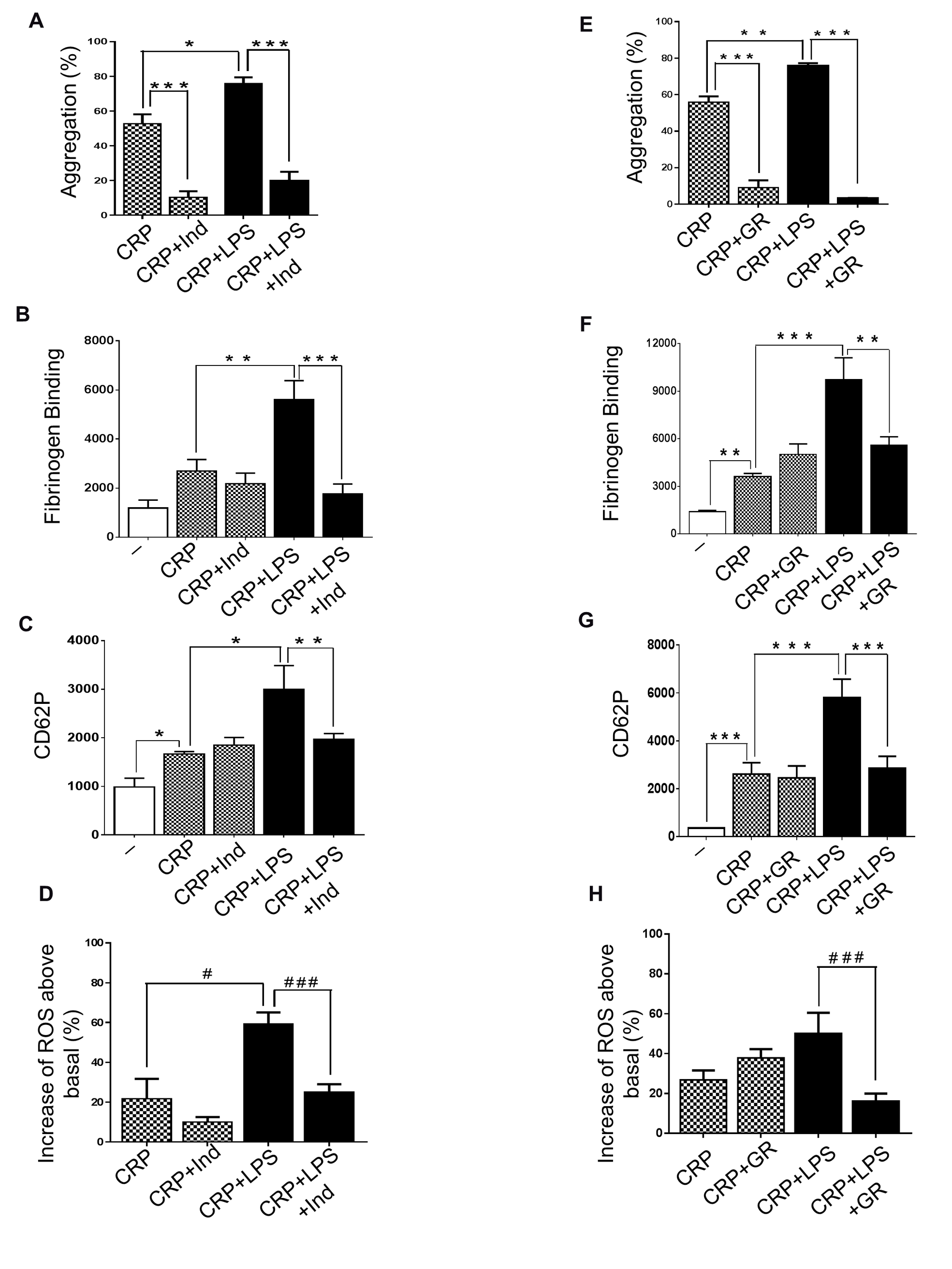

Supplement: S5 Fig — Aggregation of human washed platelet was measured by optical aggregometry following stimulation with CRP-XL (0.25μg/mL) in the presence or absence of LPS from E. coli O111:B4 (7.5g/mL) after 3 min of incubation with Indomethacin (10μM) or GR32191 (100ng) (A and E). The effect of CRP-XL (0.25μg/ml) and LPS- on fibrinogen binding and P-selectin exposure after incubation with indomethacin (10μM) or GR32191 (100ng) were measure in PRP by flow cytometry (B, C, F and G). Washed platelets (4 x 10^8^/mL) were pre-incubated with DCFHDA (10μM) in the presence or absence of Indomethacin (10μM) or GR32191 (100ng) before being stimulated with CRP-XL (0.25μg/ml) in the presence or absence of LPS from E. coli O111:B4 (7.5μg/mL) and ROS levels were analysed by flow cytometry (D and H). Cumulative data represent mean values ± SEM (n = 4). (Anova-Bonferroni test, * P≤ 0.05; ** P≤ 0.01; *** P≤ 0.001; Test t student # P≤ 0.05; # # # P≤ 0.001). (TIF) [file pone.0186981.s005.tif]
